# Desmoid-type fibromatosis of the head and neck in children: a case report and review of the literature

**DOI:** 10.1186/s13256-016-0949-9

**Published:** 2016-06-10

**Authors:** Hidetaka Miyashita, Seiji Asoda, Tomoya Soma, Kanako Munakata, Masaki Yazawa, Taneaki Nakagawa, Hiromasa Kawana

**Affiliations:** 1 Division of Oral and Maxillofacial Surgery, Department of Dentistry and Oral Surgery, School of Medicine, Keio University, 35 Shinanomachi, Shinjyuku-ku, Tokyo 160-8582 Japan; 2 Department of Plastic and Reconstructive Surgery, School of Medicine, Keio University, 35 Shinanomachi, Shinjyuku-ku, Tokyo 160-8582 Japan

**Keywords:** Desmoid, Fibromatosis, Children, Head and neck, Decision-making, Tongue, Case report

## Abstract

**Background:**

Desmoid-type fibromatosis is defined as an intermediate tumor that rarely occurs in the head and neck of children. There is no doubt as to the value of complete surgical excision for desmoid-type fibromatosis. However, in pediatric patients, surgeons may often be concerned about making a wide excision because of the potential for functional morbidity. Some studies have reported a lack of correlation between margin status and recurrence. Therefore, we discussed our findings with a focus on the state of surgical margins.

**Case presentation:**

We report an unusual case of a 9-month-old Japanese girl who prior to presenting at our hospital underwent debulking surgery twice with chemotherapy for desmoid-type fibromatosis of the tongue at another hospital. We performed a partial glossectomy and simultaneous reconstruction with local flap and achieved microscopic complete resection. We also reviewed available literature of pediatric desmoid-type fibromatosis in the head and neck.

**Conclusions:**

We described successful treatment for the refractory case of pediatric desmoid-type fibromatosis. The review results showed that some microscopic incomplete resections of tumors in pediatric patients with desmoid-type fibromatosis tended to be acceptable with surgical treatment.

## Background

According to the World Health Organization’s classification of head and neck tumors [1], desmoid-type fibromatosis (DF) is defined as a borderline tumor of soft tissues that has low malignant potential. DF is characterized by local aggressiveness with an approximate 20 % local recurrence rate, but without metastasis [1]. The annual incidence of DF is presumed to be 0.2 to 0.4 per 100,000 individuals [2]. Among cases of DF, 7 to 15 % of tumors occur in the head and neck [1, 3, 4]. A paper on the European Organisation for Research and Treatment of Cancer (EORTC)/Soft Tissue and Bone Sarcoma Group’s position on DF reported that a “watch and wait strategy” is the first choice in the treatment of DF in all populations and that resection with a clear margin should be considered to be a treatment option if postoperative morbidity is acceptable [5]. Although complete resection (negative microscopic margin; CR) of the tumor is thought to be the reference standard for successful treatment in patients with DF uncontrolled by other treatment approaches, resection in the head and neck region is often difficult because of the presence of vital structures [6]. This problem is worse in pediatric patients. In pediatric patients with DF uncontrolled by non-surgical treatments, surgeons sometimes are concerned about performing wide resections because of the high potential of postoperative morbidity. When a large tumor exists close to a vital structure, surgery with an adequate safety margin may be challenging. Although some successful cases with incomplete resection or without surgery have been reported [7–12], a randomized trial of treatment strategies has not yet been reported.

In our view, many surgeons have limited experience on how to decide on performing ablation in pediatric patients with DF. Thus, we reviewed the available literature to determine various factors that could influence treatment decisions in pediatric DF. In particular, we focused on the correlation between margin status and disease condition and if there are differences in disease condition between microscopic incomplete resection (microscopic positive margin but no remaining gross tumor; MIR) and gross incomplete resection. As part of our study, we included a case involving a 9-month-old Japanese girl with DF of the tongue who was surgically treated at our oral and maxillofacial surgery division.

## Case presentation

**Fig. 1:**
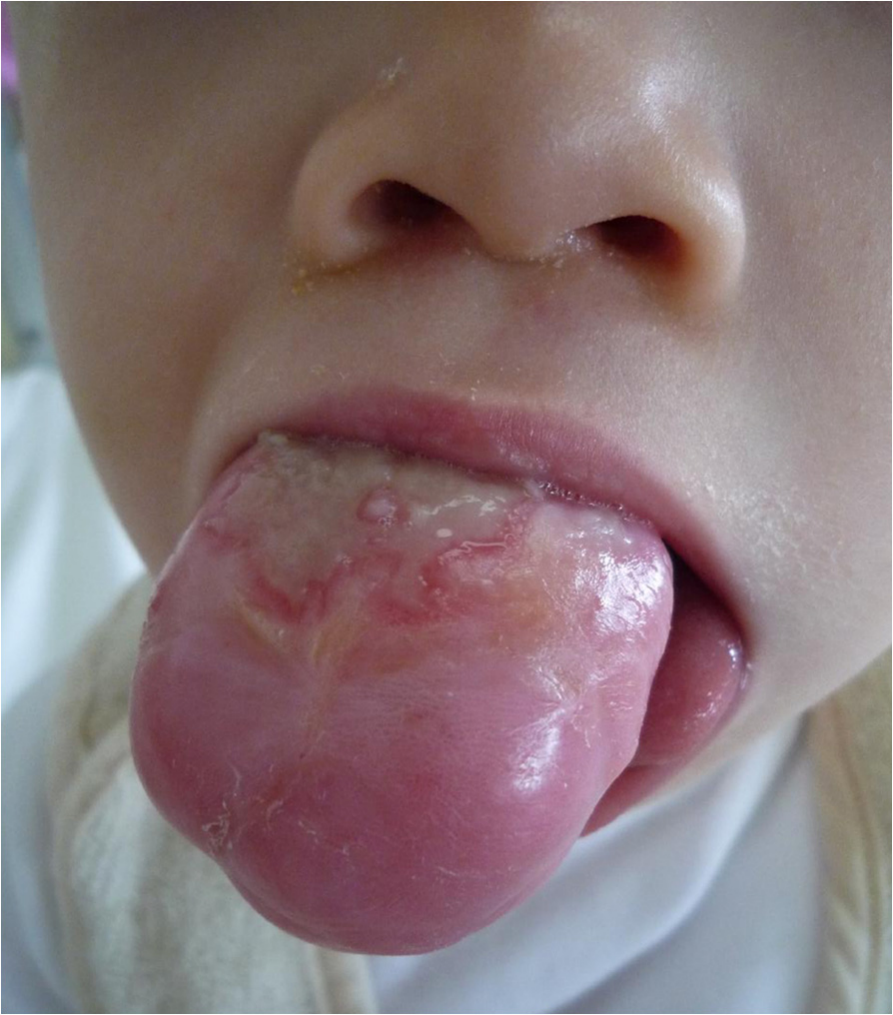
The patient could not close her mouth completely because of the presence of a part of the tumor outside her mouth. The maximum tumor size reached 5 cm

**Fig. 2:**
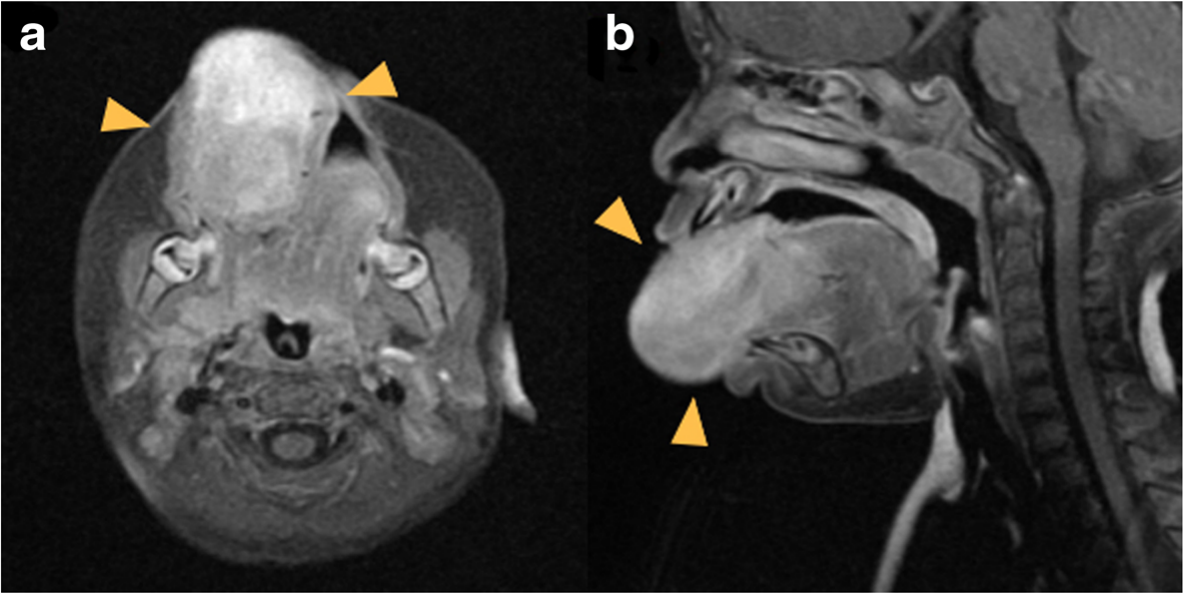
**a** Magnetic resonance imaging (MRI) showing huge mass with contrast enhancement in the right side. **b** MRI of saggital plane did not show the posibility of infiltration into the root of the tongue. Arrows indicate the areas of tumor with contrast enhancement

**Fig. 3:**
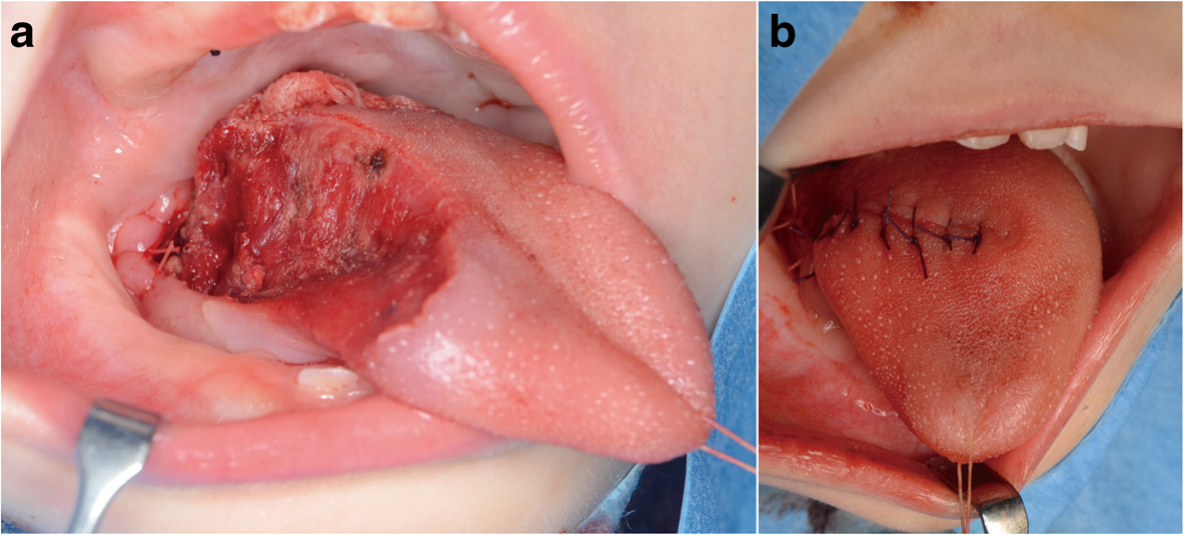
**a** A partial glossectomy with a 5-mm safety margin was performed. **b** We performed simultaneous reconstruction with a local flap and rotation of the anterior tongue on the unaffected side into the tongue defect on the affected side

**Fig. 4:**
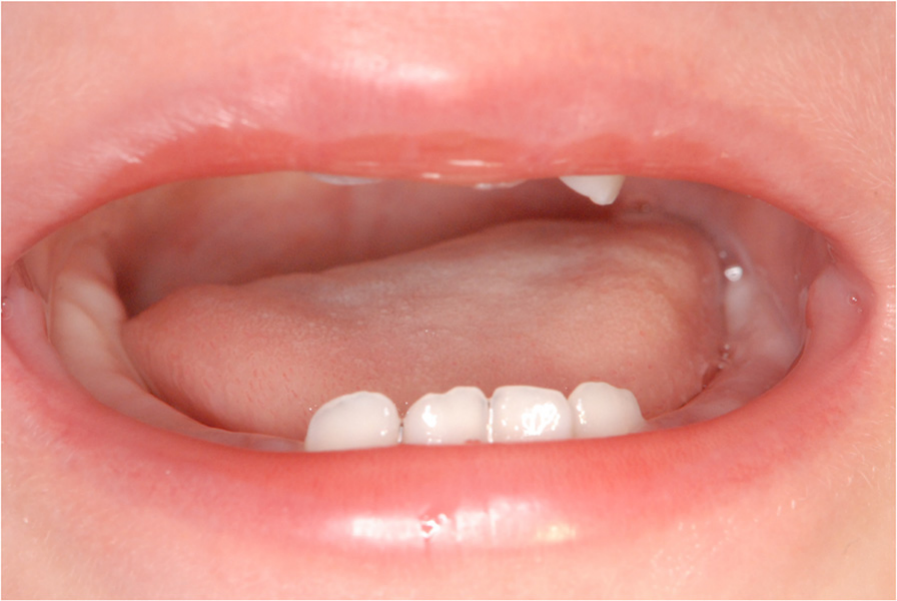
Intraoral findings after a year postoperatively

A 9-month-old Japanese girl was referred to our hospital because of a growing tumor on her tongue. Her family noted the mass in her tongue at the age of 3 months. She twice underwent debulking surgery with medical treatment, involving NSAIDs, vinblastine plus methotrexate, and vinblastine plus actinomycin-d at another hospital. The analysis of a resected specimen led to the diagnosis of DF at the age of 7 months. However, the tumor was not controlled. When she was brought to our hospital, the tumor rapidly increased, and a portion popped out of her mouth (Fig. 1). She could not close her mouth completely, but swallowing dysfunction and upper respiratory infection was not observed. Her swallowing function was complemented by trick motion of the unaffected side. She had no history of any disorders associated with DF. A whole body computed tomography examination revealed no signs of any other tumoral lesions. On the basis of radiological examinations and the clinical course, we made the decision to perform radical surgery. Magnetic resonance imaging (MRI) with contrast enhancement showed the mass (Fig. 2). A partial glossectomy with a 5-mm safety margin and simultaneous reconstruction with a local flap were performed under general anesthesia (Fig. 3). After excision, primary wound closure was performed without any graft while being careful to preserve postoperative function. We confirmed a microscopic CR in a pathological examination of the surgical specimen. Her postoperative progress was extremely good, and no functional morbidity, such as eating dysfunction or dysphonia, was observed. She had no indication of recurrence after a year (Fig. 4).

## Discussion

We performed a review of the literature by searching PubMed and using the keywords “desmoid,” “fibromatosis,” and “pediatric.” We found 97 articles from 1982 to 2015 when we searched cross-sectionally. The established exclusion criteria were as follows: details in individual cases not described, patients who were >19-years old, reports not written in English, and tumor sites not in the head and neck. We examined the following factors in all cases: age, sex, tumor site, tumor size, treatment, margin status, recurrence, complication, disease condition, and follow-up duration. The descriptive terms of incomplete resection (MIR), residual tumor, positive, and CR, which relate to margin status, were defined as follows: MIR, microscopic positive margin but no remaining gross tumor; residual tumor, remaining gross tumor; positive, positive resection margin, but we could not determine if it was MIR or a residual tumor; CR, negative microscopic margin; and NR, data not shown. The cases were reviewed in detail and are discussed in this case report.

**Fig. 5:**
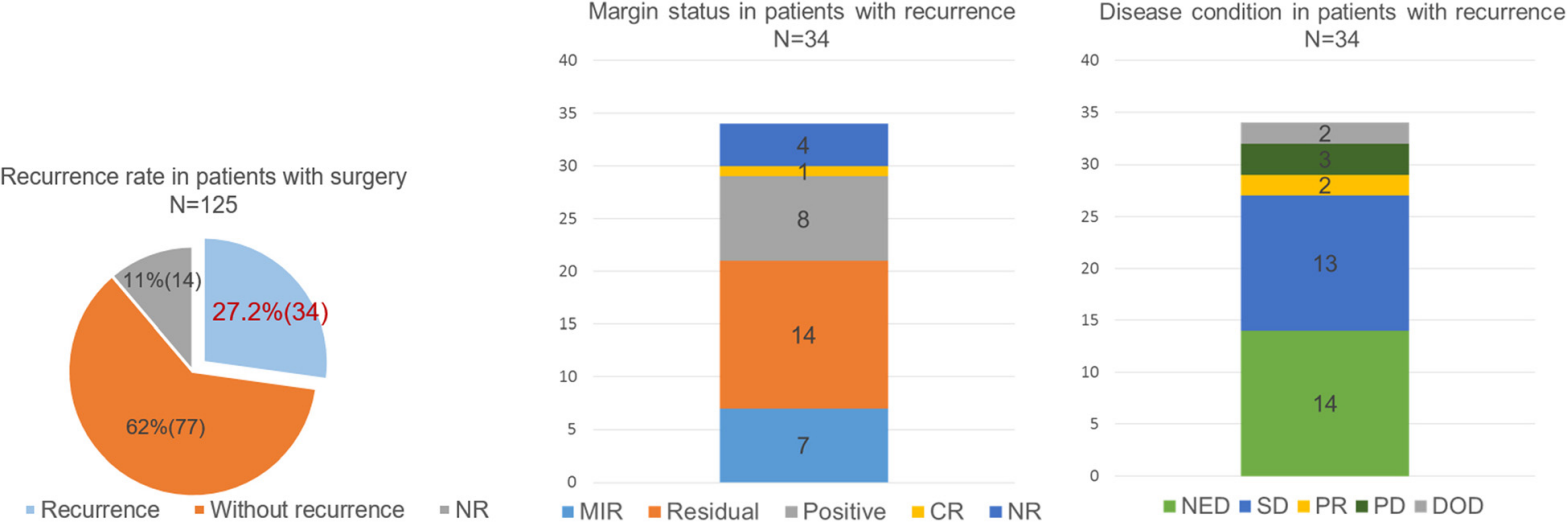
Details of margin status and disease condition in recurrent patients. *CR* complete resection, *DOD* dead of disease, *MIR* microscopic incomplete resection, *NED* no evidence of disease, *NR* no report, *PD* progressive disease, *Positive* MIR or Residual, *PR* partial response, *Residual* gross incomplete resection, *SD* stable disease

**Fig. 6:**
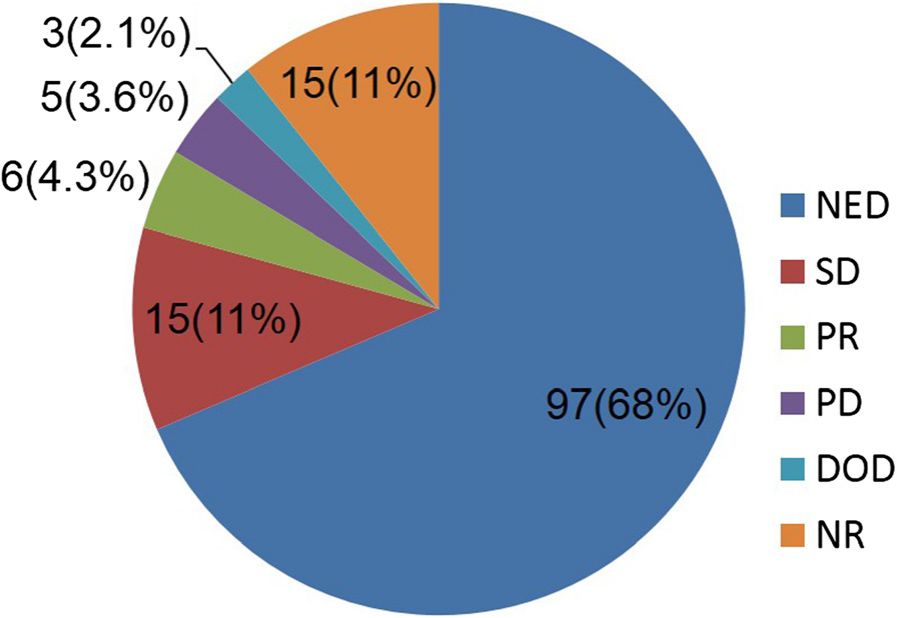
Disease condition in all patients (*n*=141). *DOD* dead of disease, *NED* no evidence of disease, *NR* no report, *PD* progressive disease, *PR* partial response, *SD* stable disease

We identified 141 patients with DF in the head and neck [7, 8, 13–72]. Age at diagnosis ranged from birth to 18 years, and the mean age was 4 years 3 months. The sex of the patient was reported in 92 % of all cases (67 males and 63 females). Regarding the tumor sites, a majority of tumors arose from the mandible (25 %). Other sites were as follows: submandibular area, infratemporal fossa, neck, peritracheal area, and paraspinal area. A list of tumor sites is presented in Table 1. We were able to identify the tumor size in 78 (55 %) patients. The average tumor size was 43.6 mm (range, 5 to 100 mm). Surgery was performed in 125 (88.6 %) patients during the treatment period, and the remaining patients were treated only with chemotherapy (12 patients), the watch and wait strategy (3 patients), or a combination of radiation and chemotherapy (1 patient). Margin status was identified in 78 (62.4 %) of the 125 patients. The number of patients who had MIR, residual tumors, positive status, and CRs was 24, 19, 19, and 16, respectively. Recurrence was observed in 34 (27.2 %) of the 125 patients with surgery. The most common margin status was residual, which was observed in 14 patients. The number of patients who had positive status, MIR, CR, and NR was 7, 8, 1, and 4, respectively (Fig. 5). Postoperative complications were identified in detail in 13 patients. Trismus was the most common complication (*n*=6). Two patients experienced secondary papillary carcinoma due to radiation therapy. Osteomyelitis (*n*=*2*), slight ptosis due to a sectioned facial nerve (*n*=1), restrictive neck movement (*n*=1), and Claude–Bernard–Horner syndrome (*n*=1) were confirmed. The disease condition was identified in 126 of the 140 patients (Fig. 6). Details were not reported for 15 patients. Ninety-seven patients showed no evidence of disease (NED). Stable disease (SD), partial response (PR), progressive disease (PD), and death occurred in 15, 6, 5, and 3 patients, respectively. The average follow-up duration was 53.3 months (range, 2 to 298 months).

**Table 1:** Primary tumor sites

Location of tumors	The number of patients
Mandible	35 (2)
Submandibular area	22 (2)
Neck	13 (5)
Tongue	12 (3)
Cervical paraspinal area	8 (7)
Infratemporal fossa	8 (3)
Parapharyngeal space	7 (1)
Maxilla	7 (2)
Peritracheal area	3 (3)
Paranasal sinus (ethmoidal, sphenoid)	3 (1)
Floor of mouth	2 (1)
Forehead skin	2
Lip	2
Parotid gland	2
Parietal	2
Nuchal area	2 (1)
Cervicothoracic	1 (1)
Suprahyoid	1 (1)
Canthus	1
Cheek	1
Hypoglossal	1
Oropharynx	1 (1)
Hypopharynx	1
Orbit	1
Periorbital	1
Submental	1
Scalp skin	1

The numbers within parentheses are the numbers of recurrences in patients with surgery

**Fig. 7:**
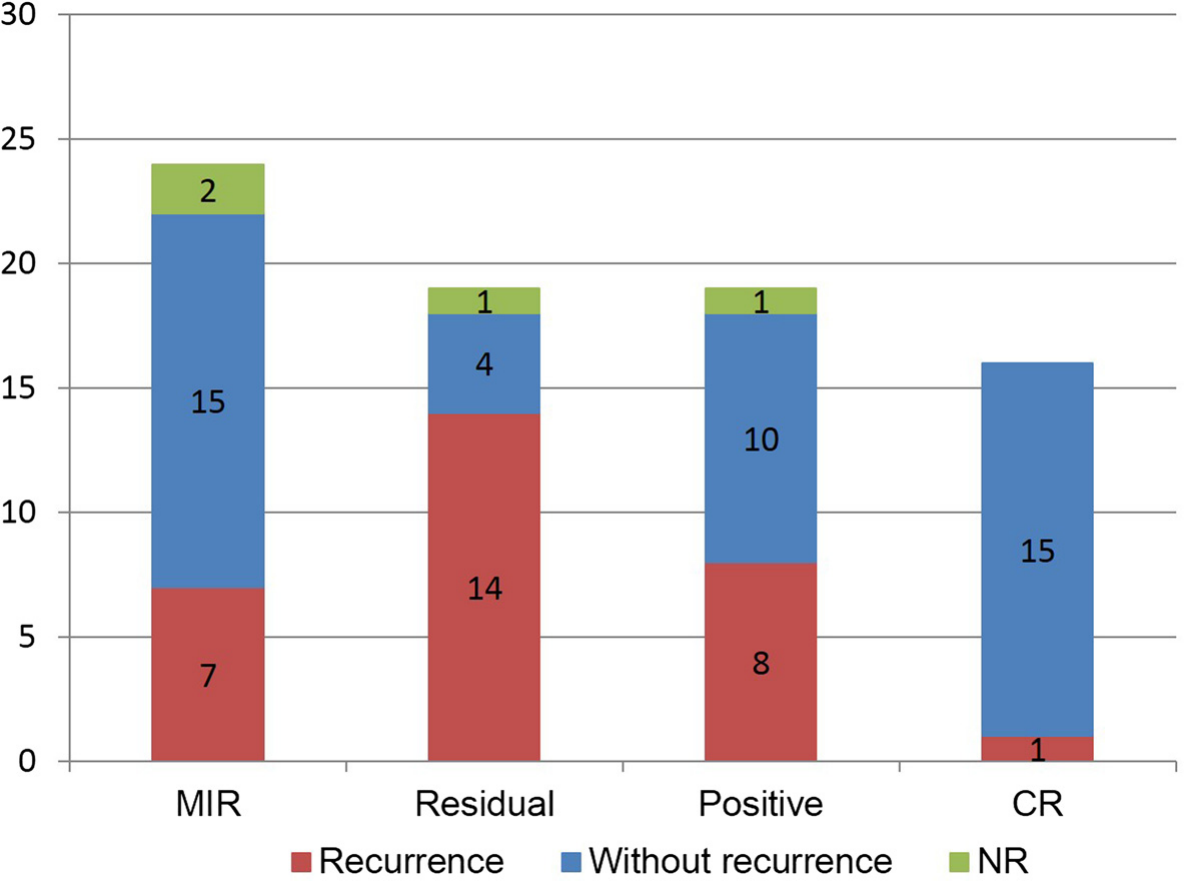
The number of recurrences for each margin status. *CR* complete resection, *MIR* microscopic incomplete resection, *NR* no report, *Positive* microscopic incomplete resection or residual gross incomplete resection, *Residual* gross incomplete resection

The most common tumor site in the recurrent patients was the cervical paraspinal region (seven patients). The next most common tumor site was the neck (five patients). Other sites we could identify were as follows: peritracheal area, infratemporal fossa, submandibular area, and tongue. All tumor sites in the recurrent patients are shown in Table 1. The recurrence rates for each margin status were MIR, 7 (29.1 %) of 24; residual, 14 (73.6 %) of 19; positive, 8 (42.1 %) of 19; and CR, 1 (6.2 %) of 16 patients (Fig. 7). There were 29 patients with positive margins without recurrence; “positive” status as used here includes MIR, residual, and positive status. Adjuvant therapies for these patients were as follows. In the case of MIR without recurrence (*n*=15), observation (*n*=9), chemotherapy (*n*=4), and radiation (*n*=2) were performed. In the case of residual tumors without recurrence (*n*=4), all patients spontaneously regressed. In positive patients without recurrence (*n*=10), observation (*n*=3), only chemotherapy (*n*=5), and administration of NSAIDs plus chemotherapy (*n*=2) were performed.

**Fig. 8:**
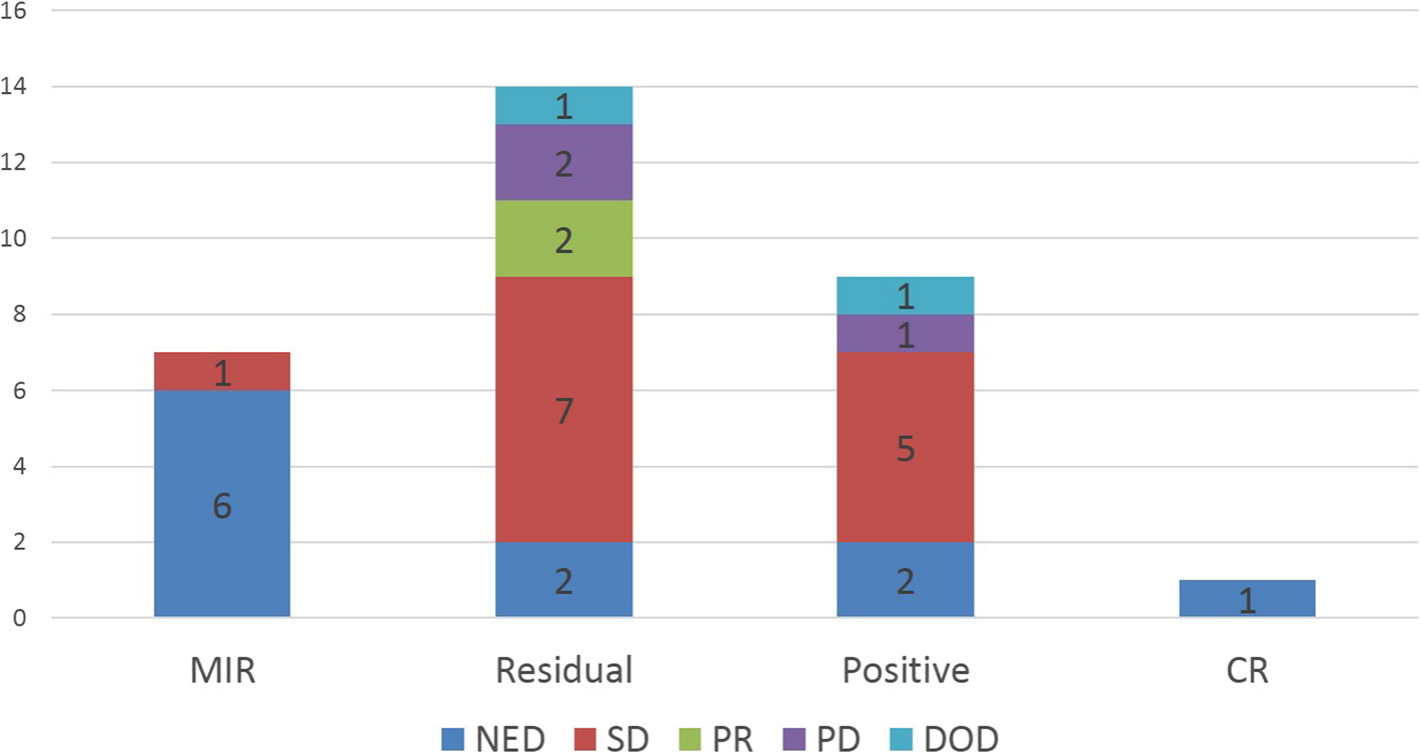
Relationship between margin status and disease condition in the recurrent patients. *CR* complete resection, *DOD* dead of disease, *MIR* microscopic incomplete resection, *NED* no evidence of disease, *PD* progressive disease, *Positive* microscopic incomplete resection or residual gross incomplete resection, *PR* partial response, *Residual* gross incomplete resection, *SD* stable disease

In recurrent patients, the relationship between margin status (*n*=31) and disease condition was confirmed as follows (Fig. 8). In the case of MIR (*n*=7), six (85.7 %) of seven patients had NED because second surgeries had been performed. One of the seven patients with SD was treated only with chemotherapy. In the case of residual tumors (*n*=14), there were two patients with NED, seven with SD, two with PR, two with PD, and one who died. Only 14.2 % of the patients showed controlled NED. In the case of positive status (*n*=9), there were two patients with NED, five with SD, one with PD, and one who died. There was NED in 11.1 % of the patients. The patient having CR with recurrence (*n*=1) had controlled NED. The patients having MIR (85.7 %) and CR (100 %) with recurrence had high rates of NED. It is notable that all recurrent patients with controlled NED were rescued by second surgery.

The number of patients for each disease condition in all recurrent patients (*n*=34) with surgery was 14 with NED, 13 with SD, 2 with PR, 3 with PD, and 2 who died (Fig. 5). Of all recurrent patients with surgery, 41.1 % had controlled NED. Consequently, in all cases in which surgery was performed, 91 (72.8 %) patients had controlled NED. The mean follow-up duration for patients with surgery was 53.3 months. The result of our literature review was summarized in Table 2.

**Table 2:** The results of literature review

Summary of literature review	
The number of patients	141
Mean age (years)	4.3
Gender	
Male	67
Female	63
NR	11
Treatment	
With surgery	125
Without surgery	16
Recurrence rate (%) in patients with surgery	27.2
Average follow-up duration (months)	53.3
Abstraction of principal information 1. In the margin positive patients (*N=*62), 29 patients did not show recurrence. After initial surgery, 16 of them did not need additional treatment. 2. In the patients having MIR, the recurrence rate (29.1 %) is lower and the NED rate (85.7 %) is higher than other margin positive status. 3. In the recurrent patients who had NED, all of them were treated with second surgery. 4. We identified postoperative complications in detail in 13 patients including radiation-induced secondary papillary carcinoma.	

*MIR* microscopic incomplete resection, *NED* no evidence of disease, *NR* no report

The main finding of our study was that the tumor control rate tended to be high in cases of MIR. Moreover, there were 29 margin-positive patients who were without recurrence. A majority of these patients had MIR (*n*=15), and 15 (62.5 %) of 24 patients with MIR did not show recurrence. In our view, these results suggest that a finding of MIR could be acceptable during local aggressive DF treatment in children. A paper on EORTC’s position on DF in all populations [5] proposed that if positive microscopic surgical margins were found at a pathological examination, no further treatment should be considered. Our review results supported this position in pediatric patients with head and neck DF. Wide resection may induce long-term functional morbidity, especially in children, and may require reconstruction. Even though microvascular reconstruction is useful in pediatric patients for well-experienced surgeons [73, 74], the decision to proceed with wide resection should be carefully considered; the results of this study may be useful when making decisions in such cases.

If progression occurs after the watch and wait strategy has been pursued, medical therapy is clearly recommended in patients with head and neck DF according to the paper on EORTC’s position [5]. However, a standard treatment is yet to be established. Previous prospective studies in pediatric patients with DF, including a phase II trial of vinblastine plus methotrexate and tamoxifen plus NSAIDs, have reported that the CR and PR rates were 19.2 % and 8 %, respectively [75, 76]. Even when CRs are included, the total response rate is insufficient. The restrictive effectiveness of chemotherapy regimens, such as liposomal doxorubicin, anthracycline-based regimen, imatinib, and sorafenib, in the total population has been confirmed [77–80]. However, no high-grade evidence study (that is, a randomized phase III trial) has been reported. In the present study, in only one patient, chemotherapy using Adriamycin (doxorubicin) and dacarbazine achieved CR. Furthermore, in the recurrent patients, there were none with controlled NED who had been treated only with subsequent chemotherapy. They were rescued by second surgery. Therefore, at present, the therapeutic effect of chemotherapy may be limited in pediatric DF treatment. A randomized trial would be needed to confirm the efficacy of chemotherapy in pediatric DF treatment.

Although previous reports have indicated that treatments with surgery and radiotherapy in all populations improve the progression-free survival rate relative to that of surgery alone [3, 81], the use of radiotherapy during DF treatment is controversial. However, in pediatric patients, radiotherapy may be less available because of late adverse effects and lower effectiveness. Side effects including secondary carcinoma related to radiotherapy for DF in pediatric and young-adult patients have been reported [53, 82]. A medium-sized retrospective study involving 30 patients in a single institution who were treated with radiotherapy for pediatric and young-adult DF reported a lower regional control rate for patients who were <18-years old (20 %) than for those who were 18 to 30-years old (63 %) [53]. In the present study, 13 patients received radiotherapy, and complications were identified in three patients: two patients developed secondary papillary thyroid cancer after total doses of 55 Gy and 50 to 60 Gy (not specified), and one developed osteoradionecrosis after a total dose of 55 Gy. There is no clearly standardized evidence-based radiation strategy for pediatric head and neck DF. Some refractory patients may need radiation therapy. However, it may not be reasonable to routinely consider radiotherapy for pediatric patients with DF having positive margins.

## Conclusions

The present study cannot provide the same level of evidence as could be obtained in a prospective study, and no single institution retrospective studies were included in the review because details were lacking. However, we believe that the collected results can be helpful in making treatment decisions, especially those involving surgery, in pediatric patients with DF. In summary, microscopic positive margins in pediatric DF did not always lead to an uncontrolled condition. In some patients, such findings appeared to be acceptable in pediatric patients with local aggressive DF. Nevertheless, further evidence-based approaches are needed before DF treatment strategies can be standardized.

## Abbreviations

CR, complete resection; DF, desmoid-type fibromatosis; EORTC, European Organisation for Research and Treatment of Cancer; MIR, microscopic incomplete resection; MRI, magnetic resonance imaging; NED, no evidence of disease; NR, data not shown; PD, progressive disease; PR, partial response; SD, stable disease
